# Investigating the Transformation Products of Selected Antibiotics and 17 α-Ethinylestradiol under Three In Vitro Biotransformation Models for Anticipating Their Relevance in Bioaugmented Constructed Wetlands

**DOI:** 10.3390/toxics11060508

**Published:** 2023-06-05

**Authors:** Lucas Sosa Alderete, Andrés Sauvêtre, Serge Chiron, Đorđe Tadić

**Affiliations:** 1 Institute of Environmental Biotechnology and Health, INBIAS-CONICET, Universidad Nacional de Río Cuarto, Ruta Nacional 36 Km 601, Río Cuarto 5800, Córdoba, Argentina; lsosa@exa.unrc.edu.ar; 2 HSM, University Montpellier, CNRS, IRD, 34090 Montpellier, France; andre.sauvetre@mines-ales.fr (A.S.); serge.chiron@umontpellier.fr (S.C.); 3 HSM, University Montpellier, IMT Mines Ales, CNRS, IRD, 30100 Ales, France

**Keywords:** emerging pollutants, transformation products, hairy root cultures, *Trichoderma*, xenobiotic metabolism

## Abstract

The degradation of three antibiotics (sulfamethoxazole, trimethoprim, and ofloxacin) and one synthetic hormone (17 α-ethinylestradiol) was investigated in three in-vitro biotransformation models (i.e., pure enzymes, hairy root, and *Trichoderma asperellum* cultures) for anticipating the relevance of the formation of transformation products (TPs) in constructed wetlands (CWs) bioaugmented with *T. asperellum* fungus. The identification of TPs was carried out employing high-resolution mass spectrometry, using databases, or by interpreting MS/MS spectra. An enzymatic reaction with β-glucosidase was also used to confirm the presence of glycosyl-conjugates. The results showed synergies in the transformation mechanisms between these three models. Phase II conjugation reactions and overall glycosylation reactions predominated in hairy root cultures, while phase I metabolization reactions (e.g., hydroxylation and N-dealkylation) predominated in *T. asperellum* cultures. Following their accumulation/degradation kinetic profiles helped in determining the most relevant TPs. Identified TPs contributed to the overall residual antimicrobial activity because phase I metabolites can be more reactive and glucose-conjugated TPs can be transformed back into parent compounds. Similar to other biological treatments, the formation of TPs in CWs is of concern and deserves to be investigated with simple in vitro models to avoid the complexity of field-scale studies. This paper brings new findings on the emerging pollutants metabolic pathways established between *T. asperellum* and model plants, including extracellular enzymes.

## 1. Introduction

Emerging pollutants (EPs), including pharmaceuticals, are of environmental concern due to their inherent bioactive properties and their persistence [1]. The main conventional method applied in wastewater treatment plants (WWTPs) for EPs removal has been ozonation [2]. Unfortunately, this technology can generate potential toxic transformation products (TPs) if a post-treatment step is missing [3]. Consequently, alternative, greener technologies such as phytoremediation or bioremediation have been considered [4]. Phytoremediation can be implemented through the development of constructed wetlands (CWs) [5]. CWs offer excellent scenarios to investigate different variables such as bacteria, fungi, substrates, plants, and their synergies to improve the remediation of contaminated water [6]. In addition, there have been specific strategies such as aeration and bioaugmentation to improve the removal of recalcitrant EPs [7,8]. Recently, a bioaugmentation assay of CW with the *Trichoderma* fungus was implemented in our research group at pilot-scale. However, due to the complexity of the treatment system and the low EPs concentrations, the elucidation of EPs transformation pathways was nearly impossible. Most of the work has been limited to studying the abatement of parent compounds and the formation of selected TPs [9]. Trying to unravel the EPs metabolic pathways requires, therefore, the use of simple in vitro biotransformation models. For this purpose, purified enzymes such as plant peroxidases (PODs) and fungal laccases have been widely applied [10]. Validated models have also included *Trichoderma* spp. strains [11,12] and in vitro hairy root cultures (HRC) [13]. The use of fungus in bioaugmentation has emerged as a safer alternative compared to bacteria, demonstrating reduced risks of antimicrobial resistance development [14]. Additionally, *Trichoderma* spp. have been widely used in agricultural applications due to their antagonistic ability against plant pathogens and their ability to regulate plant growth [15]. Similar to *Trametes versicolor*, *Trichoderma* spp. possess a machinery of unspecific enzymes (e.g., laccase and peroxidase enzymes), which makes this fungus attractive for applications in EPs removal [16]. Even though *Trichoderma* spp. and HRC have been used separately in xenobiotic removal studies, little information is available on the synergies of both biological models. Therefore, the main goals of this present paper were oriented to (i) investigate the potential ability of HRC from two plant species (*Nicotiana tabacum* and *Armoracia rusticana*) and *Trichoderma asperellum* strain *T34,* to remove selected EPs (i.e., sulfamethoxazole, trimethoprim, ofloxacin, and 17 α-ethinylestradiol), (ii) identify their respective TPs by liquid chromatography-high-resolution mass spectrometry (LC-HRMS) and elucidate their biotransformation pathways, and (iii) to compare the two in vitro biotransformation models to anticipate the relevance of potentially formed TPs in CWs bioaugmented with *T. asperellum*.

## 2. Materials and Methods

### 2.1. Reagents

Sulfamethoxazole (SMX), trimethoprim (TMP), ofloxacin (OFL), and the synthetic hormone 17 α-ethinylestradiol (EE2) were purchased from Sigma-Aldrich (St Quentin-Fallavier, France), with a purity of >95%. To evaluate the extraction efficiency of these compounds from the HRC tissue and liquid medium, the deuterated labeled compounds sulfamethoxazole-d_4_ (SMX-d_4_), trimethoprim-d_3_ (TMP-d_3_), and ofloxacin-d_3_ (OFL-d_3_) were purchased from Sigma-Aldrich (St Quentin-Fallavier, France) and used as internal standards at a final concentration of 0.1 mg/L. Methanol (MeOH), acetonitrile (ACN), and formic acid (HCOOH) were all HPLC grade (purity > 98.0%) and were purchased from Carlo Erba Reagents S.A.S (Val de Reuil, France). Milli-Q water from a Milli-Q Advantage A10 system (Sigma-Aldrich, St Quentin-Fallavier, France) was used when ultrapure water was required. All individual standard solutions and their stock mixtures were prepared with 100% (*v*/*v*) MeOH at a concentration of 1000 mg/L. 

### 2.2. Biological Material 

Plant material: Horseradish (*A. rusticana*) and tobacco (*N. tabacum*) HRCs were grown under previously established experimental conditions [17]. For this, HRC were sub-cultured each 25–30 days on Murashige and Skoog (MS) (Sigma–Aldrich, St Quentin-Fallavier, France) liquid medium supplemented with vitamins [18]. For the removal kinetic assays, the HRC were grown and maintained for 15 d under dark conditions at 25 ± 2 °C on an orbital shaker at 70 rpm.

Fungal material: *Trichoderma asperellum* strain T34 was kindly provided by the Department of Vegetable Biology, Faculty of Biology, University of Barcelona (Barcelona, Spain). *Trichoderma asperellum* was routinely grown in potato dextrose broth (PDB), obtained from Sigma–Aldrich (St Quentin-Fallavier, France), in a solid or liquid state, supplemented with previously filtered (0.22 μm PTFE syringe filter) lactic acid 0.01% (*v*/*v*) to avoid bacterial growth. *Trichoderma asperellum* was maintained by replication carried out every week with new PDB agar petri dishes at 25 °C. 

### 2.3. Emerging Pollutant Removal by HRC, T. asperellum, and Pure Enzymes

HRC (0.4 g) were grown in Erlenmeyer flasks containing 50 mL of MS medium and incubated at 25 ± 2 °C in darkness on an orbital shaker at 70 rpm for 15 days, the necessary time for the HRC to reach the exponential growth phase. HRC were treated with a solution containing 1 mg/L of a mixture of SMX, TMP, OFL, and EE2. The removal assays were carried out at room temperature. HRC were harvested in triplicate each day until the end of the experiment. For the control conditions, HRC were grown in the absence of ABs and EE2. After sampling, HRC tissue was washed twice with bi-distillated water and dried with filter paper. Samples were then turned into powder using a mortar and pestle and liquid N_2_ and stored at −80 °C for further studies. Once the HRC were sampled, the residual liquid media was collected and stored at −20 °C for further analysis. To evaluate the effect of adsorption on the HRC tissue and the abiotic degradation of targeted compounds, autoclaved HRC were fortified with tested compounds and processed in the identical way as previously described. *Trichoderma asperellum* was obtained from 8-day-old cultures was used for the in vitro removal assays. Supernatants (1 mL) obtained after centrifugation (4000× *g* rpm for 15 min) were used as inoculum in Erlenmeyer flasks containing 50 mL of PDB medium. For the EPs removal assay, 7-day-old *T. asperellum* cultures were exposed to 1 mg/L SMX, TMP, OFL, and EE2 for 1 d and 4 d. In order to induce laccase activity and increase the ability of *T. asperellum* to in vitro remove EPs, PDB medium was supplemented with 0.1 mM CuSO_4_ (Sigma–Aldrich, St Quentin-Fallavier, France), 3 days before EPs exposure as described by [19]. To avoid EP photodegradation, all removal experiments were performed under dark conditions. In vitro enzymatic reactions were carried out using purified horseradish peroxidase type IV (*HRP*) (Sigma–Aldrich, St Quentin-Fallavier, France) and *T. versicolor* laccase (*TvLacc*) (Sigma-Aldrich, St Quentin-Fallavier, France). The mixture reaction containing a final volume of 2 mL was composed of 50 mM sodium acetate/acetic acid buffer pH 5 and pH 4.5 for HRP and *Tvlacc*, respectively, using three different enzyme concentrations (i.e., 0.1, 1 and 10 U) and 1 mg/L of each EP. The reaction time was 1 and 4 h at room temperature. In the case of HRP, it was necessary to include hydrogen peroxidase (Sigma–Aldrich, St Quentin-Fallavier, France) as a co-substrate at a final concentration of 5 mM. The enzymatic reaction was stopped by the addition of 12.5 µL of 250 mM chlorhydric acid, then filtered with 0.2 µm of the PTFE filter, both obtained from Sigma–Aldrich (St Quentin-Fallavier, France), and transferred to vials ready for injection at LC-MS.

### 2.4. Extraction and Analysis of Emerging Pollutants and Their Transformation Products

Extraction: For analysis of EPs and their TPs, an extraction and sample clean-up protocol were performed for both HRC tissues and liquid culture medium (MS and PDB) according to the analytical methodology described by Campos et al. (2019) [20] with slight modifications. Briefly, 0.5 g of HRC tissue was mixed with 5 mL of methanol by vortex for 1 min, placed in an ultrasonic bath, sonicated for 10 min, and centrifuged at 5000× *g* rpm for 10 min. Supernatants were recovered in a clean tube and combined from two extraction cycles. Supernatants were then dried under a gentle nitrogen flow for complete evaporation and resuspended in 0.2 mL of MeOH (10%, *v*/*v*), sonicated by ultrasound for 5 min, filtered with 0.2 µm of the PTFE filter, and transferred to vials ready for injection. For the liquid culture medium, 10 mL of medium was diluted with 20 mL of Milli Q water and extracted by solid-phase extraction (SPE) using Oasis HLB cartridges (200 mg sorbent, 6 cc, Waters). Prior to extraction, the Oasis HLB cartridges were activated with 6 mL of methanol followed by 6 mL of Milli Q water under vacuum. Analytes were recovered by eluting the cartridges with 6 mL of methanol. These eluates were set under a gentle nitrogen flow for complete evaporation, and finally, residues were reconstituted with 0.2 mL of MeOH (10%, *v*/*v*), filtered with 0.2 µm of the PTFE filter, and transferred to vials ready for injection at LC-HRMS. A matrix match calibration curve was applied for the quantification of parent compounds. 

LC-HRMS analysis: Chromatographic separation was conducted using a Waters XBridge BEH C18 (2.1 × 150 mm and 2.5 µm particle size) column equipped with a pre-column. The chromatography assays used for the analysis of ABs involved a 10 μL injection volume, a 0.30 mL/min flow rate, and a binary gradient of water (A) and acetonitrile (B), both containing 0.1% formic acid, as follows: 10% B at 0–1 min, 90% B at 10–23 min, 10% B at 24–29 min. EE2 was analyzed using acetonitrile containing ammonium as the organic mobile phase (B) with the same gradient as previously described [21]. The column temperature was set to 30 °C. Extracts were analyzed on a HPLC Accelera 600 pump coupled to a Q-Orbitrap HRMS mass spectrometer (Thermo Fischer Scientific, Les Ullis, France) equipped with a heated electrospray ionization probe (HESI) source for detection. ABs and their TPs were analyzed in positive ionization mode, whereas EE2 and its TPs were analyzed in negative ionization mode. The HESI parameters were as follows: 40 arbitrary units (AU) sheath gas; 15 AU auxiliary gas; 300 °C capillary temperature; 200 °C heater temperature, and the electrospray voltage was set at 3.0 kV for the positive and 2.5 kV for the negative ionization modes. The S-lens radio frequency (RF) level was set at 100 AU. Full scan data were acquired at a resolution of 70,000 full width at half maximum (FWHM) with an automatic gain control (AGC) of 1 × 10^6^ 10e^6^, 250 ms of the maximum ion injection time, and a scan range of 100–1000 m/z. Moreover, for MS^2^, data-dependent acquisitions (DDA) and data-independent acquisitions (DIA) were achieved at a resolution of 17,500, two absolute collision energies (20 eV and 40 eV), an isolation window of 1 m/z, an AGC of 5 × 10^4^, 150 ms of the maximum ion injection time, and a scan range of 50–1000 m/z.

### 2.5. Determination of Plant POD and Fungal Laccase Activity

HRC (100 mg) were weighed into 1.5 mL Eppendorf-type plastic tubes, keeping the tubes on ice, and homogenized with 0.6 mL of 50 mM sodium acetate/acetic acid pH 5.5 buffer plus 1 M KCl buffer solution. Then, the samples were homogenized for 10 s and centrifuged at 3354× *g* for 10 min. The obtained supernatant was transferred to new tubes and was considered the crude extract of total PODs. POD activity was determined according to the protocol previously described [17]. For this, a mixture reaction containing 100 mM sodium acetate/acetic acid buffer, pH 5, 0.63 mM o-dianisidine (o-D) (substrate), and 0.5 mM H_2_O_2_ (oxidizing agent), and 5 μL of POD extract (diluted 1/20 with 50 mM AcNa/AcH buffer, pH 5) or 10 μL from cultured MS medium was made. In the presence of PODs and H_2_O_2_, o-dianisidine was oxidized to the colored product [bis (3,3′-dimethoxy-4-amino) azo-biphenyl], which was detected at λ = 470 by continuous spectrophotometry at 35 °C (Shimadzu UV/VIS spectrophotometer). Similarly, to analyze the *T. asperellum* laccase activity, 2 mL of a solution consisting of 50 mM of acetate buffer (AcNa/AcH) pH 4.5, 0.63 mM o-dianisidine (o-D), plus an aliquot of 0.5 mL of *T. asperellum* culture medium, as laccase source, was employed. One unit of the enzyme (U) was defined as the amount of enzyme able to generate 1 μmoL of product in 1 min in the described conditions. Reagents were purchased from Sigma–Aldrich (St Quentin-Fallavier, France).

### 2.6. Confirmation of Glycosylated Transformation Products and Indirect Quantification

To confirm in vitro glycosylation of contaminants and indirectly estimate the rate of conjugation, in vitro enzymatic reactions were performed in the presence of β-glucosidase [22]. More specifically, 0.1 mL of 50 mM sodium acetate buffer (pH 5) and β-glucosidase (5 U) were added to the tissue and culture medium extracts (obtained as above mentioned) and incubated overnight at 30 °C. The reaction was stopped by the addition of 14 µL of perchloric acid (70%, *v*/*v*), filtered with 0.2 µm of the PTFE filter, and transferred to vials ready for injection. For controlled samples, only buffer without β-glucosidase was added. Reagents were purchased from Sigma–Aldrich (St Quentin-Fallavier, France).

### 2.7. Data Analysis

A one- or two-way ANOVA was performed using STATISTICA 6.0 (StatSoft, Tulsa, OK, USA), and significant differences between treatments were calculated by Tukey’s test with a significance level of 0.05 (*p* < 0.05). Before the significance test, normality and homogeneity of variance were verified using the Shapiro–Wilk and Levene tests, respectively. If the assumption of homogeneity of variance was not confirmed, the data were transformed using an appropriate function. Data acquisition and processing were carried out using Xcalibur™ version 2.2.1 (Thermo Fisher Scientific, Les Ulis, France) with Qual and Quan browsers. To compile the list of possible TPs for further DDA, raw files from Orbitrap were uploaded to Compound Discoverer 3.1 (Thermo Fischer Scientific, San Jose, CA, USA) and processed with the Environmental Untargeted Metabolomics workflow.

## 3. Results and Discussion

### 3.1. EP Removal from the Medium and EP Bioaccumulation in Tissues

**Figure 1:**
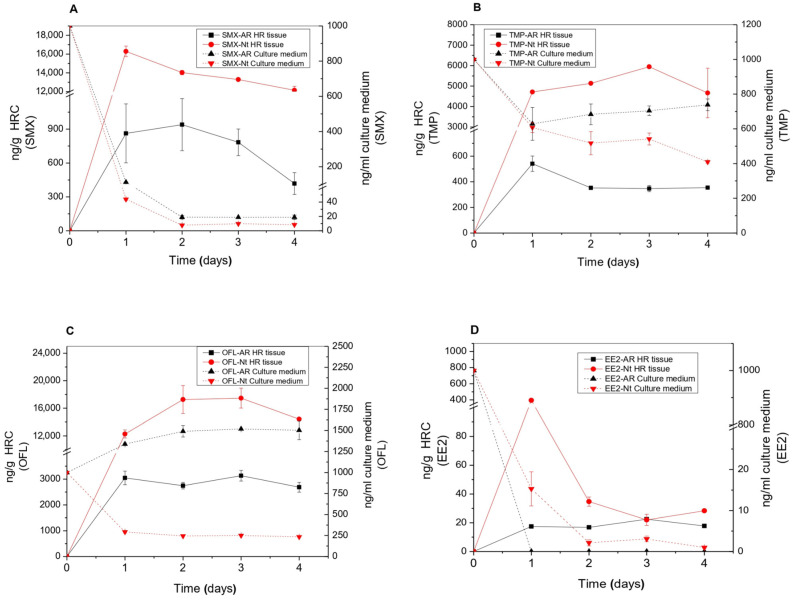
Determination of removal kinetics in residual culture medium (dash lines) and bioaccumulation within the tissue (solid lines) of SMX (**A**), TMP (**B**), OFL (**C**), and EE2 (**D**) in *A. rusticana* (AR) (black lines) and *N. tabacum* (Nt) (red lines) hairy root cultures. These hairy roots were grown for 15 days on liquid MS medium and treated with 1000 ng/mL of a mixture of these emerging pollutants for 4 days.

As a whole, horseradish and tobacco HRC showed to be very efficient in eliminating the selected EPs from the culture medium. After four days of exposure, both HRC were able to almost completely remove EE2 and SMX (Figure 1A,B,D, and Table S5). Horseradish HRC were able to remove up to 37% and 60% of TMP and OFL, respectively, whereas tobacco HRC showed percentages close to 60% TMP and 80% OFL removal (Figure 1C and Table S5). 

The SMX removal efficiencies observed in both HRC were higher than those exhibited by other plant species. For instance, SMX concentrations of 270 ± 0.03 ng/mL and 176 ± 5.23 ng/mL were detected in the culture medium of two-week-old plants and suspension cells of *Arabidopsis thaliana* after 10 and 4 days of treatment with 3000 ng/mL and 1000 ng/mL of SMX, respectively [23,24]. These amounts correspond to removal rates of 91% in the case of whole Arabidopsis plants and 82% for cell suspensions, against 99% and 98% for horseradish and tobacco HRC in our case (Figure 1A and Table S5). Studies with algae showed SMX removal rates of 40% in algae cultures exposed to 10 ng/mL for 14 days [25] and of 74% in microalgae cultures exposed to 100 ng/mL for 40 days [26]. 

For TMP, horseradish and tobacco HRC also showed similar removal percentages on the first day of exposure, 37% and 40%, respectively, which only increased up to 60% in tobacco at the end of the experiment (Figure 1B and Table S5). TMP removal rates in algae cultures also remained lower, with 11% after 14 days in algae cultures exposed to 10 ng/mL [25] and 37% after 40 days in microalgae cultures exposed to 100 ng/mL [26].

Regarding OFL removal, tobacco HRC showed percentages close to 80% after 4 days of exposure (Figure 1C and Table S5). Results reported by Singh et al. [27] indicated that duckweed (*Spirodela polyrhiza*) was more efficient since it removed 93% of OFL from the culture medium after 7 days of treatment, suggesting phytoremediation was the prime mechanism of antibiotic removal. 

In the case of the synthetic hormone EE2, our HRC removed it almost completely from the culture medium (Figure 1D and Table S5). Our results showed improved efficiency in EE2 removal in comparison with results published by other researchers using different microalgae species and exposed to 0.2 mg/L or 5 mg/L for 7 and 40 days, respectively [28,29].

**Figure 2:**
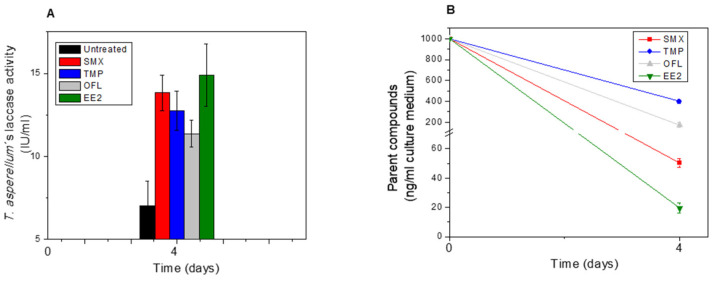
Determination of laccase activity (IU/mL) (**A**) and analysis of removal (**B**) in residual culture medium of *T. asperellum* treated with SMX (red column/lines), TMP (blue column/lines), OFL (light grey column/lines) and EE2 (green column/lines). The black column indicates an untreated condition. *T. asperellum* were grown for 7 days on DPB medium and treated with 1000 ng/mL of each emerging pollutant for 4 days.

Removal rates observed in *T. asperellum* cultures after 4 d of exposure were very similar to those found in tobacco HRC, with 98% for EE2, 95% for SMX, 80% for OFL, and 60% for TMP (Figure 2B and Table S5). Similar removal efficiencies for EE2 (98%) using *Trametes versicolor* were achieved only after a longer exposure time of 20 d [30]. The removal efficiency of SMX (95%) was higher or similar to those found in another strain of *T. asperellum* (strain BGP115) and in *Trametes versicolor*, which eliminated 71% and 90% of SMX after 7 and 10 d, respectively (Piyaviriyakul et al. 2021). The performance of *T. asperellum* to remove OFL (80%) and TMP (60%) was also higher than those obtained in previous reports using two *T. asperellum* species exposed to 200 ng/mL of OFL during 13 d (Manasfi et al., 2020). TMP was the most recalcitrant EP among the tested compounds. However, *T. asperellum* showed a higher ability to remove it from the culture medium (Figure 2B). 

The ability for EPs bioaccumulation in tissues was also evaluated in both HRC and could be ranked as OFL > SMX > TMP > EE2 (Figure 1A–D). Tobacco HRC showed higher EP accumulation than horseradish HRC, with specific differences depending on EPs and exposure time. Overall, the amount of EPs accumulated in horseradish HRC tissues was between 6 and 20 times higher than in tobacco roots. In some cases, an early significant accumulation was followed by a gradual decline in concentrations supporting further metabolism in root tissues following uptake (see, for instance, Figure 1A,D for SMX and EE2 in tobacco HRC). The bioaccumulation of EE2 in tobacco HRC was low, reaching a peak of 400 ng/g after one day. A further decrease of EE2 in tissue and culture medium suggests a very fast metabolism of the compound in plant tissue, probably due to its structural and biological similarity to plant endogenous compounds. The highest accumulation potential was detected for OFL in both HRC types. A similar accumulation pattern was also found in whole plants such as *Phragmites australis* [31]. Therefore, despite lacking transpiration as a driving force for water and nutrient absorption and transport into plant tissues, HRC can still be used as a model to study the uptake and metabolism of xenobiotics in plants.

Peroxidases and laccases are well-known enzymes for bioremediation of organic pollutants (including micropollutants typically found in municipal wastewater), participating in metabolism phase I oxidation reactions, and generating precursors of several biological conjugates and breakdown products in plants and fungi [32,33]. The ability of POD obtained from HRC to oxidize EPs has been previously studied using stopped flow spectroscopy in combination with LC-MS analysis [34]. POD activity was also evaluated in both HRC tissues and in their respective culture mediums as one of the main enzymes involved in phase I through oxidation reactions. EP exposure increased POD activity in both HRC species as well as in the culture medium, mainly after 1 or 4 days of treatment (Figure S1A–D). Similar results were also found in *P. australis* exposed to dual SMX and OFL treatment (1 mg/L), which significantly increased the POD activity mainly within the tissue [31]. Interestingly, POD activity increased continuously in the growing medium during the incubation time, while the removal of EPs was, in some cases, almost depleted. As shown in Figure 2A, laccase activity detected in the residual culture medium of *T. asperellum* showed a significant increase with EPs exposure (1000 ng/mL), which was twice as high under SMX and EE2-treatment, followed by TMP (1.8 fold-times) and OFL (1.6 fold-times) than the control (untreated condition). In this context, laccase activity could be responsible for EE2 and SMX removal, as was demonstrated in previous reports using laccase from *Trametes versicolor* [35,36].

As observed in our experiments, POD and laccases can be secreted by root tissues or fungal mycelium in the culture media, participating directly in the removal and degradation of EPs prior to entering plant or fungal tissue. Thus, these enzymes can play a key role in the establishment of specific degradation pathways, first in the rhizosphere and subsequently within the plant, shaping the composition and distribution of TPs in plant tissues.

### 3.2. Analysis of Transformation Products (TPs) Produced by HRC and T. asperellum

**Figure 3:**
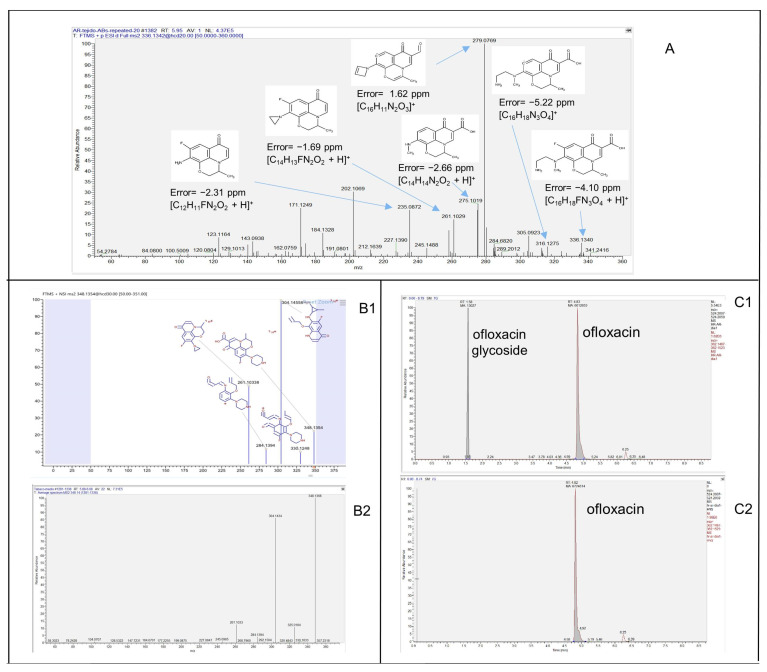
Examples of three different approaches for the identification of biotransformation products. (**A**)—identification is based on the fragmentation pattern and on the interpretation of MS^2^ spectra; (**B1**,**B2**)—identification is based on the match of MS^2^ spectra of an unknown compound with MS^2^ spectra available in the online databases; (**C1**,**C2**)—identification is based on the enzymatic reaction with β-glucosidase.

To identify the TPs obtained after biological treatment, a suspect list of possible TPs and metabolites was compiled according to the common phase I and II reactions included in the Compound Discoverer feature. In addition, other previously identified plant endogenous conjugation molecules, such as sesquiterpene lactones (e.g., lactucin, lactucopicrin, and their derivates), stress response molecules (e.g., abscisic acid), amino acids (e.g., pterin, methylsalicylate), and sugars (cladinose), were also included [23,37,38,39]. All compounds from the suspected list detected in the full-scan chromatograms were subjected to further confirmation based on their DDA-MS^2^ spectrum. Online databases such as m/z cloud (https://www.mzcloud.org/ (accessed on 27 October 2022)) and MassBank (https://massbank.eu/MassBank/ (accessed on 14 November 2022)) already contain MS^2^ spectra of some biotransformation products. However, due to the limited availability of analytical standards, these are rather limited cases. In the presented study, 5 out of 30 TPs (i.e., SMX294, OFL376-a, OFL348, TMP305, and TMP307) were identified by matching their MS^2^ spectra with the online databases (Figure 3B1,B2). For the rest of the compounds, the assessment of their molecular structures was based on the interpretation of MS^2^ spectra (Figure 3A) and application of an enzymatic reaction (β-glucosidase). 

Analysis of the TP-based diagnostic evidence obtained from MS^2^ spectra is a crucial technique, even though it is a time-consuming process. For 10 compounds, confirmation level 2b according to the Schymanski scale was reached [40]. That is to say, for these compounds, sufficiently rich MS^2^ spectra were obtained, which provided enough evidence to suggest their probable molecular structure. Moreover, an enzymatic reaction with β-glucosidase was used to confirm the presence of glycosyl-conjugates and reach level 2b. Conjugates were typically identified by revealing the presence of the parent compound’s m/z value and some of the main characteristic fragments in the MS^2^ spectra of conjugated metabolites. The observed mass difference between SMX416, TMP453, OFL524, and EE2-457 and their respective parent compounds was 162.0253, which corresponded to the neutral loss of a hexose (Tables S1–S4). In the case of SMX578 and EE2619, the mass difference with the parent compounds indicated two hexoses (Tables S1 and S4). Which hexose was present in the metabolite molecule could not be determined solely on the examination from the MS^2^ spectra. However, it is known that glucose is involved in phase II metabolization reactions in plants [41]. Since the enzymatic reactions are specific, the absence of peaks of conjugated compounds in the chromatogram and the increase of the area of parent compounds in the extracts where the enzyme was added unambiguously confirmed conjugation with glucose (Figure 3C1,C2). In addition, deglycosylation revealed that OFL540 and EE2-473 underwent both hydroxylation and glycosylation. Their peaks were not detected after the enzymatic reaction was applied, and at the same time, an increase in peak areas of hydroxyl OFL and hydroxyl EE2 was observed. Finally, 15 TPs were identified with confirmation level three, that is to say, their tentative molecular structures were proposed. The combination of the specific biotransformation reaction (most common hydroxylation) and the molecular structure of the parent molecule (e.g., aromatic ring) and its functional groups led to a speculative result. For example, for TMP325, TMP321, OFL540, and OFL364, it was practically impossible to allocate the exact place of a hydroxyl group on the parent molecule (Tables S1 and S3). Additionally, positional isomers were also detected: five hydroxy EE2 (EE2-311 a, b, c, d, and e) (Table S4) and two OFL metabolites with additional double-bonded oxygen (OFL376-b and OFL376-c) (Table S3). As mentioned, specific functional groups of the parent molecule may or may not hinder reaching higher identification confidence for their biotransformation products. Such an example is TMP, which contains three methoxy groups. We observed that demethylation and demethoxylation reactions yielded the formation of TMP277 and TMP261, respectively, but we could not distinguish the exact position, among three possibilities, at which these transformations took place (Table S2). Although LC-HRMS is the main workhorse for structural elucidation, a new hybrid approach that combines MS and nuclear magnetic resonance (NMR) spectroscopy improves the identification of unknown compounds [42,43].

Awareness about the formation and occurrence of TPs has been raised in the last decade; hence, the number of identified TPs using HRMS is increasing. Although their identification is the first and crucial step, it does not provide any information about their relevance in terms of their concentration and accumulation patterns. Evaluation of their formation kinetics is a valuable approach that can bridge this knowledge gap. 

**Figure 4:**
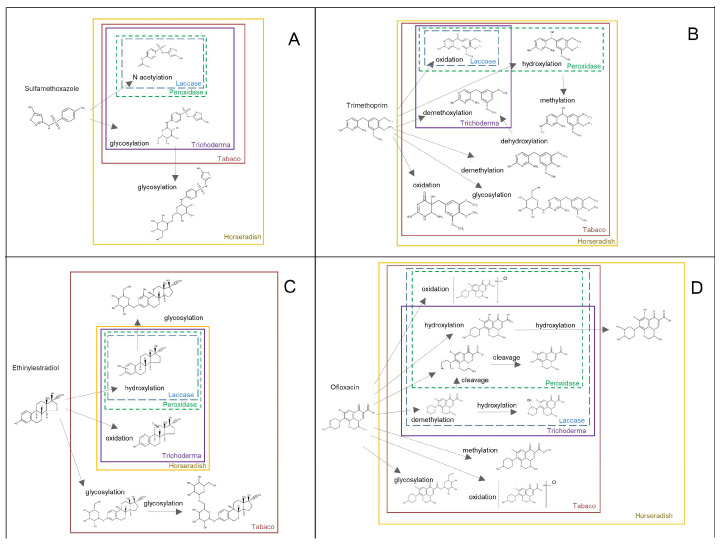
Identified biotransformation products of four parent compounds ((**A**)—sulfamethoxazole, (**B**)—trimethoprim, (**C**)—EE2, and (**D**)—ofloxacin) formed by laccase, peroxidase, *T. asperellum*, tobacco hairy roots, and horseradish hairy roots, and their respective biotransformation reactions.

**Figure 5:**
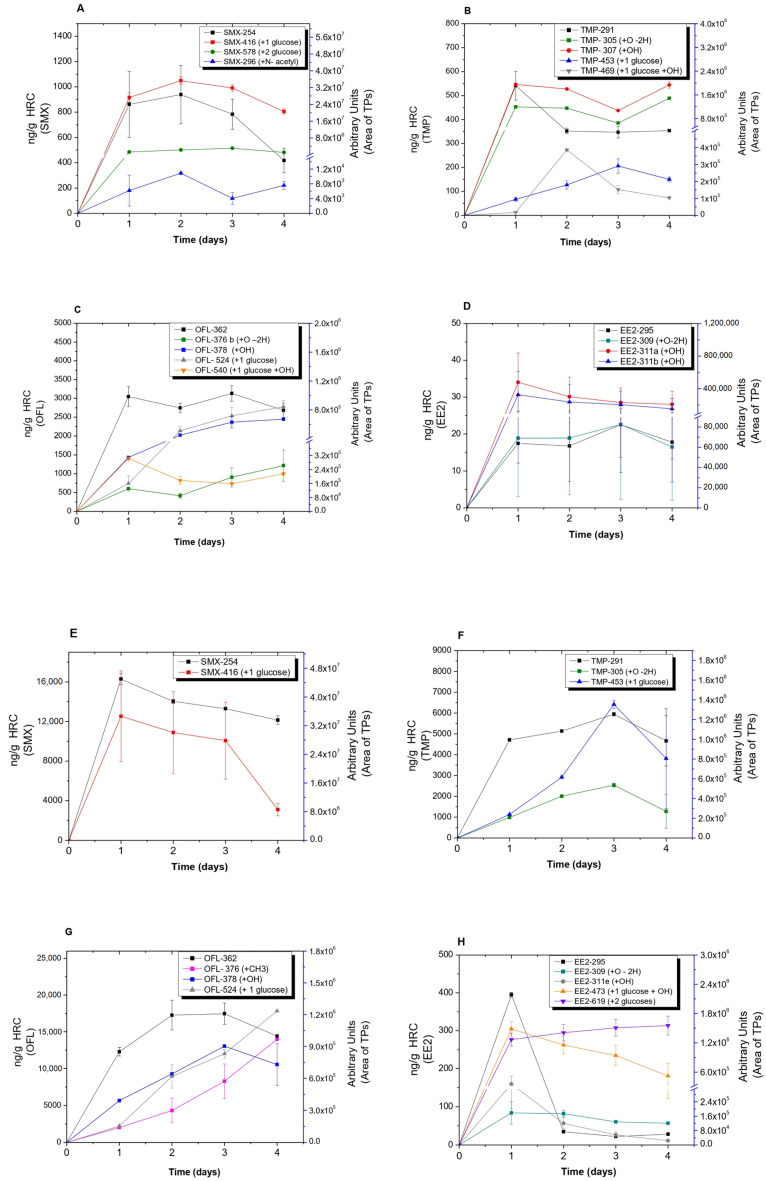
Analysis of transformation products detected in the tissue of *A. rusticana* (**A**–**D**) and *N. tabacum* (**E**–**H**) hairy roots after 4 d of treatment with a mixture of SMX, TMP, OFL, and EE2, respectively (spiking level: 1000 ng/mL of each compound).

Analyzing TPs from Sulfamethoxazole (SMX): Three TPs of SMX resulting from N-acetylation (SMX296) and glycosylation (SMX416 and SMX578) reactions were identified (Figure 4A and Table S1). The kinetics of the accumulation of detected TPs in horseradish HRC did not show any significant changes over the course of days. However, in both HRC cultures, the glycosylated SMX416 TP showed a decrease at the end of treatment, which coincided with the decrease of the parent compound within the tissue (Figure 5A,E). N-acetylation of SMX is rather known and well-studied [44], and as shown in this study, it was the compound in common for all five different experimental setups. *T. asperellum* and both plant species were able to perform glycosylation of SMX. (Figure 4 and Figure S2A). Moreover, in the case of horseradish, the consequential addition of another glucose molecule was detected (Figure 4A). Glycosylation of SMX appeared to be a spontaneous reaction as glycosylated SMX was observed in abiotic control, as well. The abiotic formation of N-acetyl and glycosylated sulfamethazine (stating the chemical affinity of sulfamethazine and glucose present in the growing media) was previously reported [23]. Due to the aforementioned, we could conclude that glycosylation is the main biotransformation pathway, as phase II can be performed directly, skipping the phase I activation reactions, due to the presence of a functional group on the parent SMX. Similarly, glycosylation was the main transformation pathway of SMX in *Arabidopsis thaliana* plants; moreover, glycosylation of glycosyl-SMX was also reported [23]. All three detected biotransformation products are of environmental concern. The antimicrobial activity might seem inactivated, but it has been shown that N-acetyl SMX can be transformed back into a parent compound [45], while SMX416 and SMX578 can release a parent compound in the presence of a suitable enzyme [46]. 

Analysis of TPs from Trimethoprim (TMP): Oxidation and hydroxylation of TMP were the most common biotransformation reactions performed in both HRC (Figure 4B), which is in accordance with previous studies [26]. The kinetics of the formation of the detected TPs showed differences in accumulation trends. For instance, a slight decay of TMP305 and TMP307 was observed in horseradish HRC after 3 days of exposure, while the glycosylated TMP469 and TPM453 increased after 2 and 3 days of exposure in both HRC (Figure 5B,F), with TMP305 and TMP453 being the only TPs detected in tobacco HRC (Figure 5F). TMP305 was detected in all experimental setups, specifically as a product of laccase activity, while TMP307 was associated with peroxidase activity (Table S2). In addition to oxidation, *T. asperellum* was able to perform demethoxylation (Figure S2B). While methylation, demethylation, consecutive hydroxylation and oxidation, and glycosylation reactions were associated with plants’ metabolization mechanisms. Demethylation and oxidation of TMP have been reported as microbial degradation pathways. More specifically, cleavage reactions were associated with specific enzymes, while oxidation reactions were associated with unspecific enzymes such as ammonium monooxygenases [47]. Proposed biotransformation reactions are present in Figure 4B. In terms of environmental relevance, identified TPs might contribute to the overall residual antimicrobial activity, especially phase I metabolites (TMP305, TMP307, TMP277, TMP261, and TMP325), since they have an intact pyrimidine moiety and no bound bulky substitutes, such as TMP321 and TMP453, that could interfere with binding to bacterial dihydrofolate reductase, which is the mechanism of TMP antimicrobial activity [37].

Analysis of TPs of Ofloxacin (OFL): In comparison with other target compounds where pure enzymes yielded the formation of one TP for EE2 and SMX and two TPs for TMP, OFL appeared to be more susceptible to enzymatic transformations in terms of the number of TPs. As it is shown in Figure 5C, horseradish HRC was able to biotransform OFL by oxidation, hydroxylation, and glucose-conjugation reactions, forming several TPs (i.e., OFL376, OFL378, OFL524, and OFL540). Regarding their accumulation profiles, OFL378 and OFL524 increased gradually after 2 d of exposure to the end of assays, while no changes were observed for OFL376 and OFL540 (Figure 5C). Similar to horseradish HRC, tobacco HRC also showed the same trend in the accumulation patterns of OFL378 and OFL524 (Figure 5G). Moreover, OFL376 (methylated OFL) showed the same accumulation trend as the other ones and was only detected in tobacco HRC, suggesting that these EPs could induce the activity of *O*-methyl transferases, as was reported in tobacco plants under other stressful conditions [48]. Alongside oxidation and hydroxylation, demethylation and further cleavage on the methyl pyrimidine ring were detected (Table S3). Plant metabolization mechanisms again formed glycosylated metabolites as well as methylated ones. There was a slight difference observed between the two plant types, as horseradish formed OFL with double hydroxyl groups and one double-bonded oxygen, which was not detected in tobacco HRC (Table S3). Moreover, OFL364 and OFL348 were only produced as results of laccase activity being more abundant after treatment with *T. asperellum* than HRC (Table S3; Figure S2C). In terms of residual antimicrobial activity, only methylation and glycosylation might inactivate it since they react with the carboxylic group, which is crucial for the OFL antimicrobial activity [49]. However, in the case of conjugates, it is just a temporary inactivation until the parent compound is released again, as described before. All the other observed transformation reactions posed minor changes to the OFL, and all of them preserved the carboxyl group, which leads us to conclude that the mixture of TPs might contribute to the overall residual antimicrobial activity. 

Analysis of TPs from 17 α-ethinylestradiol (EE2): Hydroxylation of EE2 was typical for laccase and peroxidase. Although five structural isomers for hydroxyl EE2 were observed, only two of them were relevant due to their relatively high abundance compared with other isomers (EE2-311 a–e) (Table S4). In addition to hydroxylation, *T. asperellum* and both HRC are involved in the oxidation of EE2 (Figure 4D and Figure S2D). TPs detected in horseradish HRC, such as EE2-309 and EE2-311a and b, showed an unchanged accumulation kinetic over the course of days (Figure 5D). In contrast, in tobacco HRC, hydroxylated and hydroxylated/glycosylated derivatives of EE2 (i.e., EE2-311e and EE2-473) showed the same accumulation kinetic, being the highest at the first day of exposure and decaying gradually towards the end of the experiment, whereas no changes in the accumulation trend of EE2-309 and EE2-619 were detected during the 4 d of treatment (Figure 5H). Observed biotransformation was also reported as being involved in the bacteria’s biodegradation of endocrine disrupting compounds. Surprisingly, glycosylation was not observed in horseradish samples but only in tobacco samples (Figure 4). In tobacco HRC, not only glycosyl-EE2 but also glycosyl-hydroxy-EE2 and glycosyl-glycoside-EE2 were detected. The absence of conjugated metabolites in horseradish could be attributed to the fast degradation of the parent compound in comparison with tobacco HRC. Namely, EE2 was below detection limits in culture media within 24 h (Figure 1). A comparison of the peak areas of five identified hydroxyl EE2s and their accumulation patterns revealed two structural isomers of higher relevance in tobacco HRC. The absence of structural isomers of hydroxyl glycosylated EE2 leads us to conclude there was one hydroxyl EE2 that was the most reactive and susceptible to further metabolization. 

### 3.3. Confirmation of Glucose Conjugated-TPs and Indirect Quantification by Beta-Glucosidase Assays

**Figure 6:**
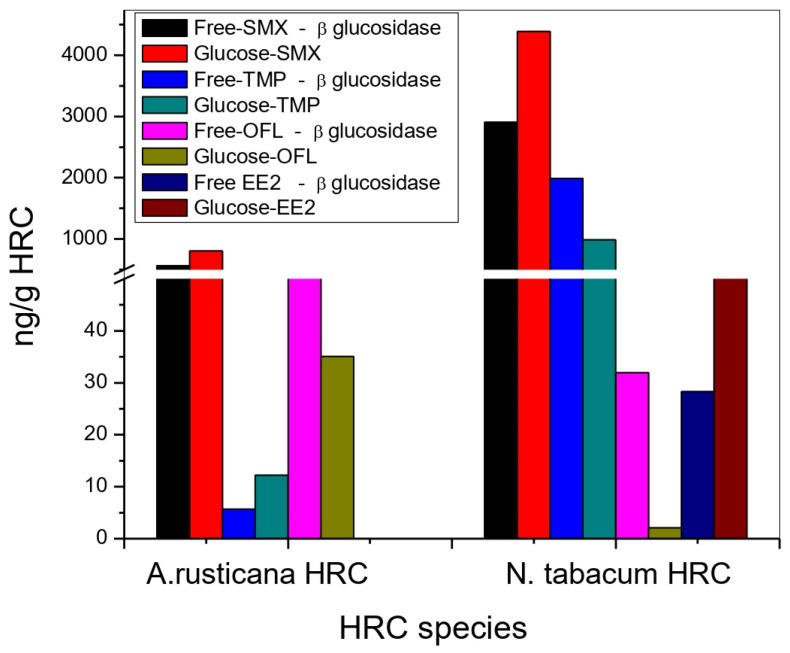
Determination of conjugated transformation products with glucose and free-parents in the presence or absence of the β-glucosidase enzyme, respectively. This assay was performed in *A. rusticana,* and *N. tabacum* hairy root cultures treated with a mixture of SMX, TMP, OFL, and EE2 (spiking level: 1000 ng/mL of each compound) during 4 d.

Glycosylation reactions, as a part of xenobiotic metabolization, were confirmed in both HRC types. As presented in Figure 5, tobacco HRC conjugated all the EPs with glucose, whereas EE2-glucoside was not detected in horseradish HRC. High peroxidase activity in horseradish HRC (50,000 IU/mL) (Figure S1A) and susceptibility of EE2 could explain the absence of glycosylated EE2. Namely, free EE2 could not be detected in the culture medium after 24 h (Figure 1D); hence, the fast degradation of EE2 in the culture medium prevented its direct conjugation. Estimated concentrations of glucose-conjugated TPs are presented in Figure 6. 

Conjugated fractions of EE2 and SMX account for 185% and 151%, respectively, expressed as a percentage of the mass fraction of the quantified free parent compound. TMP-glucoside was estimated to be 50%, whereas OFL-glucoside was only 7% in tobacco HRC. For the horseradish HRC, the highest conjugation rate was observed for TMP (216%) and SMX (143%), while the estimated conjugation rate for OFL (18%) was the lowest. N-glycosylation was observed to be the main transformation pathway of SMX in *Arabidopsis thaliana* plants, where glycosyl-SMX accounted for more than 80% of the extractable metabolites [23]. The presented results demonstrate that the bioconcentration factor (estimated as a ratio between the concentration of the target compound in, e.g., plant tissue and the concentration of the compound in the environment, e.g., water or soil) in most cases is underestimated since it does not include conjugated fractions of the parent compound, especially where relatively high uptake goes alongside fast metabolization.

## 4. Conclusions

Mechanisms involved in EP removal/transformation in real-scale nature-based solutions are rather complex, and it is practically impossible to distinguish between transformation pathways. This study provided insight into the possibilities of using HRC and *T. asperellum* fungus as in vitro biotransformation models to anticipate the relevance of TPs in more complex wetland treatments bioaugmented with fungi. Even though precise degradation pathways depended on compound-plant/fungus (system) combinations, a general conclusion could be drawn. Glysolylation reactions were the most predominant transformation pathways of the four targeted EPs in HRC, while reactions with *T. asperellum* were limited to oxidative reactions. In all cases, the persistence of residual biological activity (e.g., antimicrobial activity) could be confirmed. Studies that have only included the identification of TPs have significantly contributed to the knowledge of TPs chemical structures. However, their prioritization at this stage has been highly speculative. Due to the lack of analytical standards, it has been difficult to quantify them and test their biological activity (e.g., antimicrobial activity). In this work, analysis of TP formation/degradation kinetics during the transformation of parent compounds could provide insight into their accumulation patterns over several days. In this way, identifying the more relevant TPs became easier. Moreover, in the case of the identification of several structural isomers, as was shown for EE2, based on their formation/degradation kinetics, it was found that two out of five had relatively higher peak areas, showing their prevalence over the other three. Finally, we could not detect structural isomers for the hydroxyl-conjugated EE2, which indicated that out of 5-hydroxy-EE2, only one was further conjugated. All these results make the in vitro biotransformation models appealing to predict the formation of TPs in novel techniques applied as nature-based solutions such as enzymatic immobilization, bioaugmentation, and selection of specific plants.

## Data Availability

Not Applicable.

## Supplementary Materials

The following supporting information can be downloaded at: https://www.mdpi.com/article/10.3390/toxics11060508/s1, Table S1. Identification of SMX biotransformation products; Table S2. Identification of TMP biotransformation products; Table S3. Identification of OFL biotransformation products; Table S4. Identification of EE2 biotransformation products; Table S5: Average EPs removal rates (in %) in tobacco and horseradish HR (Nt-HR and AR-HR) and *Trichoderma asperellum* strain T34 (*Ta* T34) cultures after 4 days of incubation (*n* = 3); Figure S1. POD activity determined in tissue and culture medium of *A. rusticana* (A and B) and *N. tabacum* HR (C and D). HRCs of 15 d of growth on MS medium and were then treated with 1 ppm (1000 ng/mL) of a mixture of emerging pollutants (SMX, TMP, OFL, and EE2). Light and dark grey columns indicate untreated and treated HR cultures with the emerging pollutants, respectively; Figure S2. Transformation products detected in the culture medium of *T. asperellum* (A, B, C, and D) after 4 d of SMX, TMP, OFL, and EE2 treatment (1 ppm).
