# Supplementary material for: Investigating the Transformation Products of Selected Antibiotics and 17 α-Ethinylestradiol under Three In Vitro Biotransformation Models for Anticipating Their Relevance in Bioaugmented Constructed Wetlands
Source: Toxics. 2023 Jun 5;11(6):508. doi: 10.3390/toxics11060508 (PMC10301577; doi:10.3390/toxics11060508)

Supplementary Material

Investigating the Transformation Products of Selected Antibiotics and 17 α-Ethinylestradiol under Three In Vitro Biotransformation Models for Anticipating Their Relevance in Bioaugmented Constructed Wetland

Lucas Sosa Alderete [1], Andrés Sauvêtre [2,3], Serge Chiron [2] and Đorđe Tadic [2,*]

Instituto de Biotecnología Ambiental y Salud, INBIAS-CONICET, Universidad Nacional de Río Cuarto, Argentina, Ruta Nacional 36 Km 601, Río Cuarto CP 5800, Córdoba, Argentina; lsosa@exa.unrc.edu.ar

[2] HSM, University Montpellier, CNRS, IRD, 34090 Montpellier, France; andre.sauvetre@mines-ales.fr (A.S.); serge.chiron@umontpellier.fr (S.C.)

[3] HSM, University Montpellier, IMT Mines Ales, CNRS, IRD, 30100 Ales, France

* Correspondence: tadicdjordje86@gmail.com

Number of Tables: 5

Number of figures: 2

Table S1. Identification of SMX biotransformation products

Compound	RT (min)	m/z (error)	Molecular formula	Characteristic fragments	Molecular structure	Identification confidence (structural modification)	Laccase and peroxidase	Trichoderma	Tobacco		Horseradish	
									tissue	media	tissue	media
SMX	6.3	254.0601 (0.70 ppm)	C10H11N3O3S	188.0811<br>156.0106<br>108.0443	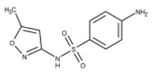	1						
SMX416	4.9	416.1117 (-0.51 ppm)	C16H21N3O8S	336.0714<br>254.0586<br>216.1229	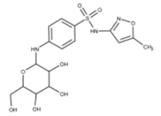	2b<br>(+ glucose - H2O)		x	x	x	x	x
SMX578	2.6	578.1640 (0.31 ppm)	C22H31N3O13S	416.1078<br>336.0712<br>254.0580		2b<br>(+ 2 glucose - 2(H2O))					x	x
SMX296	6.4	296.0703 (1.20 ppm)	C12H13N3O4S	198.0215<br>134.0598	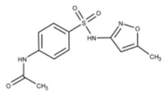	2a<br>(N-acetylation)	x	x	x		x	x

Table S2. Identification of TMP biotransformation products

Compound	RT (min)	m/z (error)	Molecular formula	Characteristic fragments	Molecular structure	Identification confidence (structural modification)	Laccase and peroxidase	Trichoderma	Tobacco		Horseradish	
									tissue	media	tissue	media
TMP	4.6	291.1452 (1.54 ppm)	C14H18N4O3	275.1143<br>261.0975<br>230.1153<br>123.0659	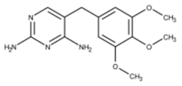	1						
TMP305	5.3	305.1250 (0.55 ppm)	C14H16N4O4	275.0755<br>244.0936<br>137.0459	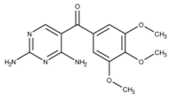	2a<br>(+ O - 2H)	x	x	x	x	x	X
TMP307	3.7	307.1406 (-1.74 ppm)	C14H18N4O4	289.1299<br>274.1059<br>259.0820	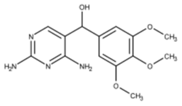	2a<br>(+ OH)	Only peroxidase			x	x	x

TMP277	6.0	277.1312 (0.75 ppm)	C13H16N4O3	261.0994 247.0814 123.0660	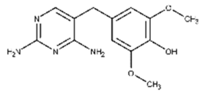	3 (- CH3)			X	x		x
TMP261	4.4	261.1352 (0.55 ppm)	C13H16N4O2	245.1032 136.0756 123.0667	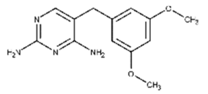	3 (- CH3O)		x		x		x
TMP325	4.1	325.1506 (0.02 ppm)	C14H20N4O5	181.0858 143.0566	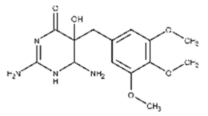	3 (+ 2OH)				x		x
TMP321	5.2	321.1545 (-1.23 ppm)	C15H20N4O4	289.1289 274.1054 259.0821	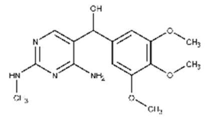	3 (+ CH3 + OH)			x	x	x	x
TMP453	4.2	453.1964 (-0.85 ppm)	C20H28N4O8	435.1856 357.1559 291.1448	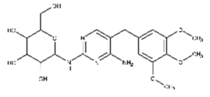	2b (+ glucose - H2O)			x		x	x

Table S3. Identification of OFL biotransformation products

Compound	RT (min)	m/z (error)	Molecular formula	Characteristic fragments	Molecular structure	Identification confidence (structural modification)	Laccase and peroxidase	Trichoderma	Tobacco		Horseradish	
									tissue	media	tissue	media
OFL	4.8	362.1505 (-1.43 ppm)	C18H20FN3O4	318.1608 261.1030	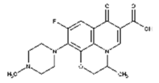	1						
OFL378	5.2	378.1458 (-0.51 ppm)	C18H20FN3O5	361.1427 317.1528 247.0874	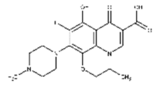	2b (+ OH)	x	x	x	x	x	X
OFL394	4.5	394.1399 (-2.54 ppm)	C18H20FN3O6	376.1303 231.0765 176.0704	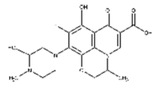	2b (+ 2OH)	x					x

OFL336	4.7	336.1340 (-4.10 ppm)	C16H18FN3O4	316.1275<br>279.0769<br>261.1029	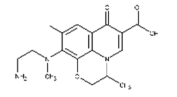	2b<br>(- C2H4)	x	x	x	x	x	x
OFL279	6.3	279.0777 (0.6 ppm)	C13H11FN2O4	261.0675<br>238.0384<br>191.0248	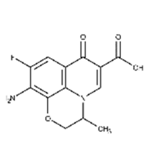	2b<br>(- methyl pyrimidine ring)	x	x	x	x	x	X
OFL-524	1.6	524.2033 (-1.09 ppm)	C24H30FN3O9	362.1501<br>318.1606<br>233.1492	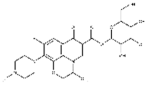	2b<br>(+ glucose - H2O)			x		x	
OFL540	4.9	540.1973 (-2.77 ppm)	C24H30FN3O10	363.1534<br>261.1033	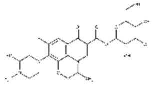	3<br>(+ glucose - H2O + OH)						
OFL376-a	4.1	376.1660 (-0.64 ppm)	C19H22FN3O4	362.1507<br>319.1087<br>305.0930	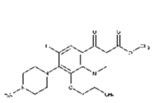	2a<br>(+ CH3)			x		x	
OFL376-b	1.5	376.1299 (-1.26 ppm)	C18H18FN3O5	358.1215<br>213.1019<br>188.0702	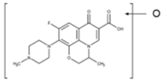	3<br>(+O - 2H)			x		x	
OFL376-c	6.5	376.1292 (0.70 ppm)	C18H18FN3O5	358.1215<br>213.1019<br>188.0702	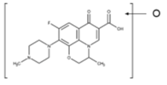	3<br>(+O - 2H)	x					x
OFL364	4.4	364.1293 (1.99 ppm)	C17H18FN3O5	346.1191<br>279.0794<br>229.0747	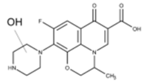	3<br>(+OH - CH3)	Only laccase	x				x
OFL348	4.6	348.1368 (3.9 ppm)	C17H18FN3O4	325.0184<br>304.1434<br>261.1033	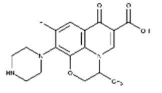	2a<br>(- CH3)	Only laccase	x	x	x	x	X

Table S4. Identification of EE2 biotransformation products

Compound	RT	m/z							Tobacco	Horseradish

	(min)	(error)	Molecular formula	Characteristic fragments	molecular structure	Identification confidence (structural modification)	Laccase and peroxidase	Trichoderma	tissue	media	tissue	media
EE2	8.7	295.1703<br>(2.46 ppm)	C20H24O2	277.1598<br>269.1556<br>159.0815<br>145.0659	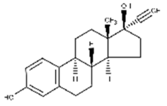	1					x	
EE2-311-a	5.5	311.1661<br>(2.67 ppm)	C20H24O3	293.1539<br>209.0973<br>195.0816<br>145.0659	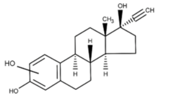	3<br>(+ OH)			x			
EE2-311-b	6.6	311.1661<br>(2.67 ppm)	C20H24O3	293.1539<br>209.0973<br>195.0816<br>161.0609<br>145.0659		3<br>(+ OH)	x		x	x		
EE2-311-c	6.8	311.1661<br>(2.67 ppm)	C20H24O3	293.1539<br>209.0973<br>195.0816		3<br>(+ OH)	Only laccase	x		x		
EE2-311-d	7.2	311.1661<br>(2.67 ppm)	C20H24O3	293.1539<br>209.0975<br>195.0816<br>175.0768<br>145.0659		3<br>(+ OH)	x		x	x	x	x
EE2-311-e	7.9	311.1661<br>(2.67 ppm)	C20H24O3	293.1541<br>209.0973<br>195.08116<br>175.0768<br>161.0609		3<br>(+ OH)		x	x		x	x
EE2-309	7.5	309.1498<br>(0.52 ppm)	C20H22O3	291.1966<br>171.1035<br>137.0973<br>119.0875	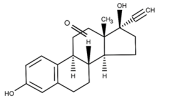	3<br>(+O - 2H)		x	x	x	x	x
EE2-457	6.9	457.2231<br>(-0.17 ppm)	C26H34O7	337.1798<br>295.1727<br>267.1393<br>133.0247	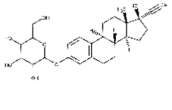	2b<br>(+ glucose - H2O)			x	x		
EE2-619	6.1	619.2782<br>(3.55 ppm)	C32H44O12	457.2252<br>323.0996<br>295.1724	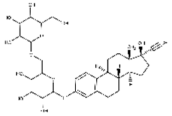	2b<br>(+ 2 glucose - 2H2O)			x	x		

EE2-473	6.6	473.2175<br>(2.66 ppm)	C26H34O8	311.1654<br>171.1035<br>128.0355	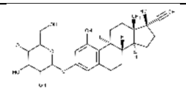	3<br>(+ OH + glucose - H2O)			x	x		

Table S5: Average EPs removal rates (in %) in tobacco and horseradish HR (Nt-HR and AR-HR) and *Trichoderma asperellum* strain T34 (*Ta* T34) cultures after 4 d of incubation (n = 3).

	AR-HR	*Nt*-HR	*Ta* T34
SMX	98	99	95
TMP	37	60	60
OFL	-	77	80
EE2	100	99.8	98

Figure S1. POD activity determined in tissue and culture medium of *A. rusticana* (A and B) and *N. tabacum* HR (C and D). HRCs of 15 d of growth on MS medium and then were treated with 1 ppm (1000 ng/mL) of a mixture of emerging pollutants (SMX, TMP, OFL and EE2). Light and dark grey columns indicate untreated and treated HR cultures with the elerging pollutants, respectively.

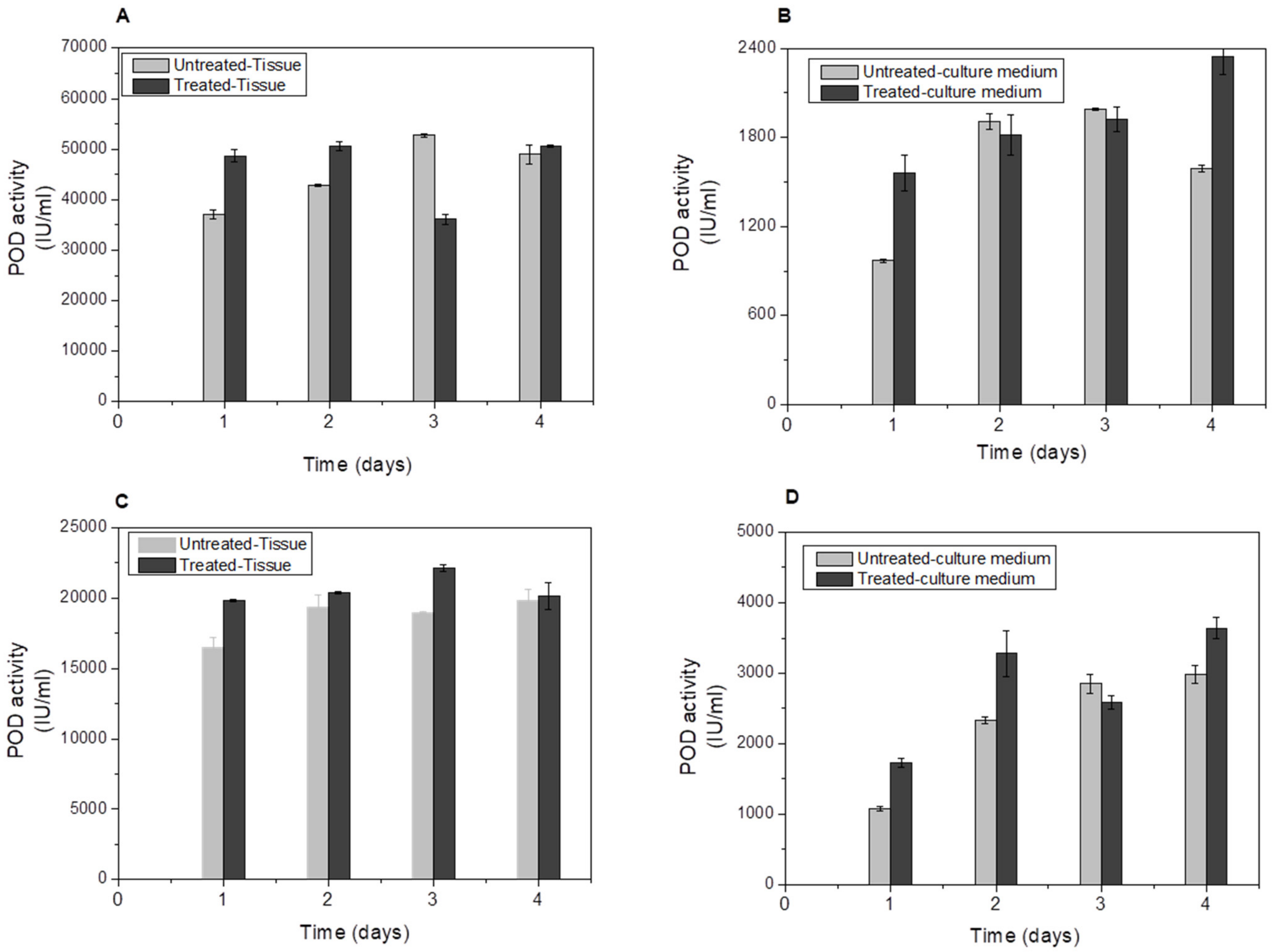

Figure S2. Transformation products detected in the culture medium of *T. asperellum* (A, B, C and D) after 4 d of SMX, TMP, OFL and EE2 treatment (1 ppm).

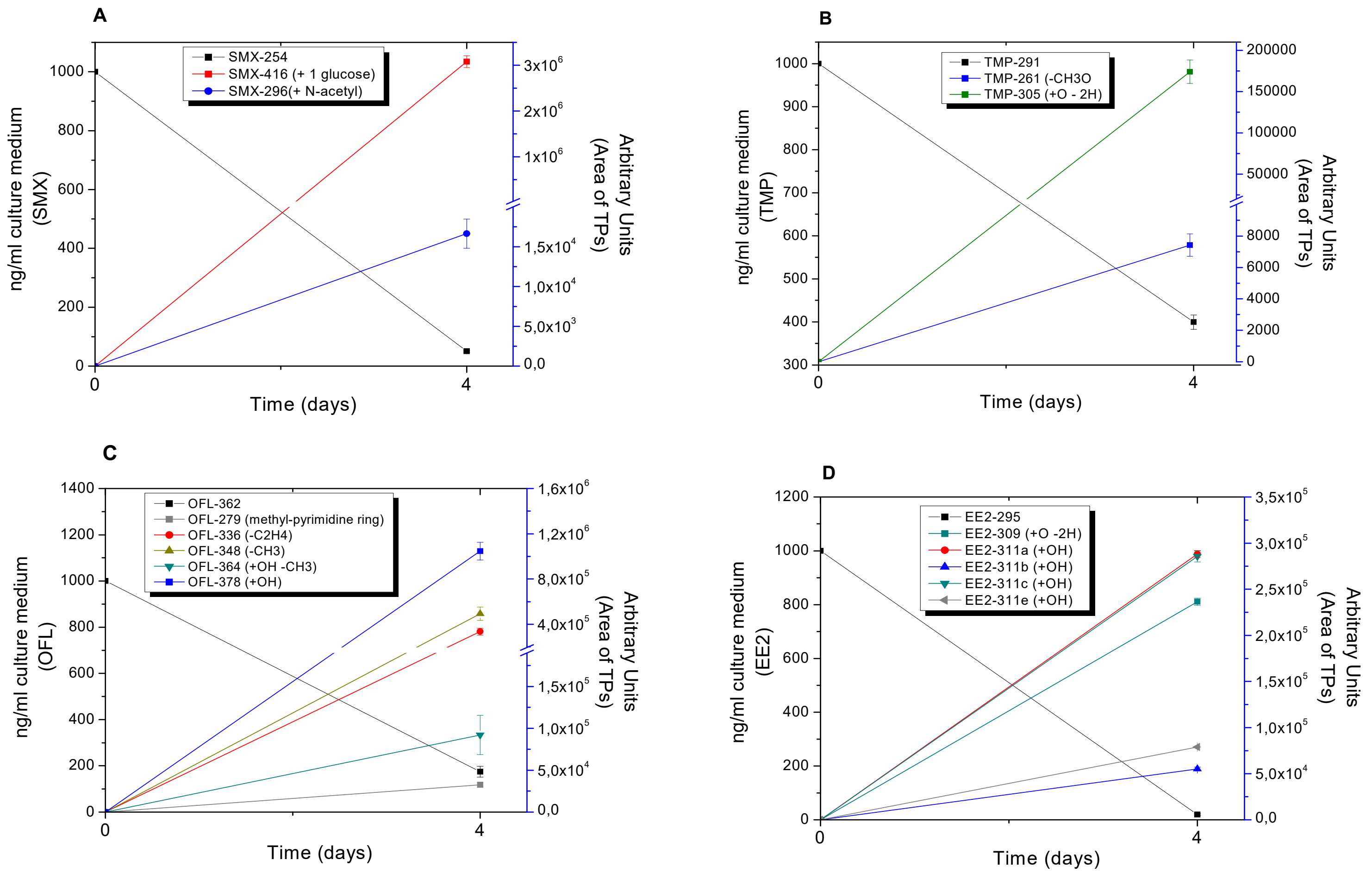

Supplement: Supplementary file 1 [file toxics-11-00508-s001.zip › toxics-2390547-supplementary.pdf]
